# Favorable Outcome and Survival Using a Hypothermia Protocol After Successful Cardiopulmonary Resuscitation

**DOI:** 10.14740/jocmr2186w

**Published:** 2015-06-09

**Authors:** Ozgur Sogut

**Affiliations:** Department of Emergency Medicine, School of Medicine, Bezmialem Vakif University, Istanbul, Turkey. Email: osogut@bezmialem.edu.tr

## To the Editor

Use of mild therapeutic hypothermia to goal 32 - 34 °C, after return of spontaneous circulation (ROSC) in cardiac arrest patients, has been shown to increase the rates of survival and favorable neurological outcomes [1, 2]. The American Heart Association (AHA) recommends mild therapeutic hypothermia after ROSC for out-of-hospital cardiac arrest with an initial documented rhythm of ventricular fibrillation (class I, level of evidence B) [3]. International resuscitation guidelines also support mild therapeutic hypothermia as the best medical practice for shockable rhythms in patients resuscitated from cardiac arrest due to ventricular fibrillation and pulseless ventricular tachycardia after ROSC [4].

Herein, we wish to notify the readers on a case of therapeutic hypothermia after ROSC in a young man associated with favorable neurologic outcome and survival. A 34-year-old electrical worker is brought to the emergency department (ED) by co-workers having been injured by electrical strike at work. On arrival in the ED, he had a Glasgow coma score (GCS) of 3/15 and dilated sluggish pupils. Additionally, there were no heartbeats or respiration. Cardiac monitoring revealed ventricular fibrillation and the patient received treatment with 150-J biphasic waveform shock. Cardiopulmonary resuscitation (CPR) was immediately instituted after the shock. After 2 min of CPR, ROSC was achieved and his rhythm has restored to sinus rhythm. Neither epinephrine nor antidysrhythmic agents were administered. He was resuscitated succesufully but yet remained comatose after CPR at the ED, following vital signs: GCS 8, heart rate 110/min, and blood pressure 80/50 mm Hg. Advanced cardiac life support measures were initiated (i.e., controlled intravenous fluid support, administration of dopamine, adequate oxygenation, and support of respiration with bag ventilation) following ROSC in the ED. Furthermore, therapeutic hypothermia protocol was started immediately with ice packs in the ED to decrease the patient’s temperature below 34 °C. Two hours after admission to the ED, he was transferred to the intensive care unit (ICU) for optimal care. Therapeutic cooling was maintained for 24 h by gel-circulation cooling pads (temperature management system; Artric Sun^®^ 5000) in the ICU, and the patient was discharged home with mild neurologic deficit on ICU day 20.

Therapeutic hypothermia is associated with favorable neurologic outcome and survival in patients who have experienced cardiac arrest and resuscitated succesufully, as it was in the present case. This therapy should be started as quickly as possible in post-cardiac arrest care patients, particularly with shockable cardiac arrest rhythms.
